# Leaf venation, as a resistor, to optimize a switchable IR absorber

**DOI:** 10.1038/srep31611

**Published:** 2016-08-24

**Authors:** M. E. Alston, R. Barber

**Affiliations:** 1 University of Salford, Manchester, UK; 2 Science and Technology Facilities Council, Daresbury, UK

## Abstract

Leaf vascular patterns are the mechanisms and mechanical support for the transportation of fluidics for photosynthesis and leaf development properties. Vascular hierarchical networks in leaves have far-reaching functions in optimal transport efficiency of functional fluidics. Embedding leaf morphogenesis as a resistor network is significant in the optimization of a translucent thermally functional material. This will enable regulation through pressure equalization by diminishing flow pressure variation. This paper investigates nature’s vasculature networks that exhibit hierarchical branching scaling applied to microfluidics. To enable optimum potential for pressure drop regulation by algorithm design. This code analysis of circuit conduit optimization for transport fluidic flow resistance is validated against CFD simulation, within a closed loop network. The paper will propose this self-optimization, characterization by resistance seeking targeting to determine a microfluidic network as a resistor. To advance a thermally function material as a switchable IR absorber.

Nature uses vascular formations to control delivery of nutrients, removal of waste, temperature regulation and damage repair by a functional fluid. A leaf is an independent unit within a tree canopy structured system that is regulated by solar orientation for daylight capture. Each leaf is a individual photosystem that is defined by rule based geometry, canopy volume, total leaf area density and angular distribution of leaf surfaces^1^, Beer’s law Fig. 1.

Vascular patterns are development mechanisms that are the mechanical support systems used by nature^2^. This is centered on fluidic metabolic material flow in cold and warm-blooded venal networks. Microchannels conform to hierarchical order by rules of minimum energy loss, minimum effective power flow rates and minimum pressure drop. This characterization will advance a thermal functional polymer as a switchable IR absorber. Thermal switching is achieved by fluidic flow to advance a photoabsorptive polymer.

In cold phloem leaves vascular patterns are characterized by the intimate relationship between vein leaf patterns and leaf foliage scale, as determined by auxin provascular activity^3^. These are highly regulated with species-specific vascular pattern formations. Vascular formations have uniform spacing patterns and exhibit spatial regularity by hierarchical sequence patterns in advanced leaf species^4^. The underlying mechanisms of vascularization pattern conduits are networks of constant flow conductivity distribution and pressure^5^. This reticulate closed loop geometry is formed by changes in vein thickness, vein angle divergence, redundancy functionality, stem vasculature fluidic supply and vein hierarchical order, (Fig. 2)^6^.

Venation network is a two dimensional (longitudinal pattern) of continuous, branching features. These vascular patterns form a complex hierarchical pattern for the transportation of fluidics for photosynthate mechanisms. Characterization of leaf vein formations are desirable morphology for solar modulation properties by microfluidics for transition temperature decrease in a thermally functional material.

Using a microfluidic-based network of steady state flow in multi microchannels across the material pane will advance a polymer for desired thermal functionality. Achieving a parabolic and laminar profile characterization to be obtained immediately from fluidic input into the network is significant. Manifold feed in and output channels perform significant roles to reduce turbulence, non-linear effects and shortcuts pathways to derive smooth flow^7^. All other multi microchannel geometry’s represent the thermal transport network for IR transmission temperature interface, for capture from the polymer surface pane. Structural assembly of a polymer of microfluidic-based flow will lead to the desired morphology to direct a photoabsorbing material to act as an IR stop-band block. This capture and store of energy is achieved by thermal absorbing fluidics in steady state flow at channel node. The research goal of the network is solar energy modulation efficiency using water flow as a thermal switching medium. The assembly of tailored flows in modulating volumetric flow resistance and pressure drop are functional significant in a materials ability to low phase transition temperature.

## Results and Discussion

Vein formations of primary (stem), secondary (mid, parallel, polar–circulative boundary vein) and minor veins (tertiary for localized fluidic flow) deal with specific leaf material regions^8^. This is functionality significant for material characterization, as it represents vein conduit sequences succession^9^,^10^. This working formation hierarchy is in response to tolerance to damage, water stress conditions and redundancy. The polar vein completes the network of nested conduit loops to maintain fluidic flow from stem and mid vein vasculature.

All veins diminish in size distally from fluidic input supply. This arrangement of diminishing vein size order by primary veins and secondary is the relationship to leaf apex scale^11^. Vasculature patterns are linked to material scale in the formation of conduit network geometry as they perform regulatory roles. The fluidic input and export flow within these hierarchical networks are subject to flow resistance and flow rate. Hydraulic resistance in fluidic conduits channels conform to minimum fluidic flow to achieved reduced pressure drop for fluidic flow efficiency. This is determined by hierarchical structure to minimize resistance R for optimal fluidic transport. The resistance is determined by mechanical energy when a flowing liquid is subjected to a change in direction. The parameter coefficient of this resistance is:


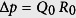



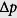
 Pressure drop between inlet and export stem vasculature

*Q* Fluidic Flow

*R* Resistance

### Vasculature as a Resistor

**Figure 3:**
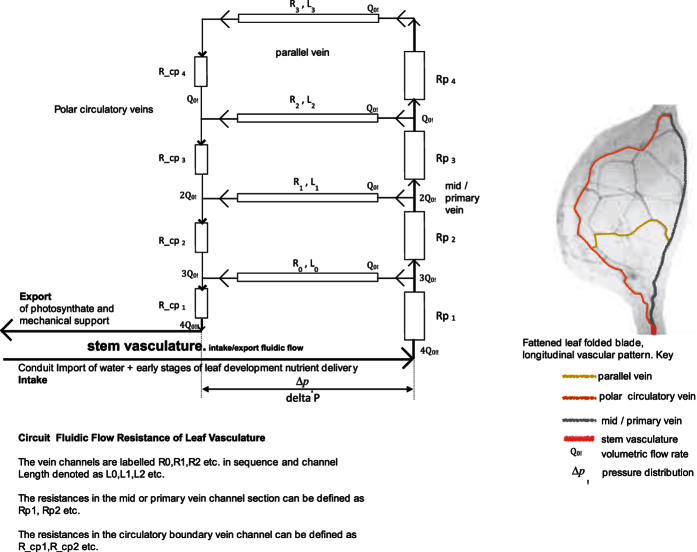
Resistor Network by Branching Network Scaling.

Optimal transport efficiency in natural fluidic pattern formations can be defined as a resistor. This is flow resistance evaluation in determining channel conduit scaling of vasculature branching networks. Channels that are distally positioned from fluidic input are affected by pressure drop in fully developed laminar flow. Veins will be subjected to increasing resistance or rather pressure drop for any given flow rate. To evaluate this hydrodynamic question essentially rules by Hagen-Poiseuille’s law, which suggests a constant flow resistance, a pressure loss linearly increasing with flow rate. Poiseuille number. (Po) can be applied to vascular leaf formations and represented as a resistor conductance circuit. Fig. 3.

**Figure 4:**
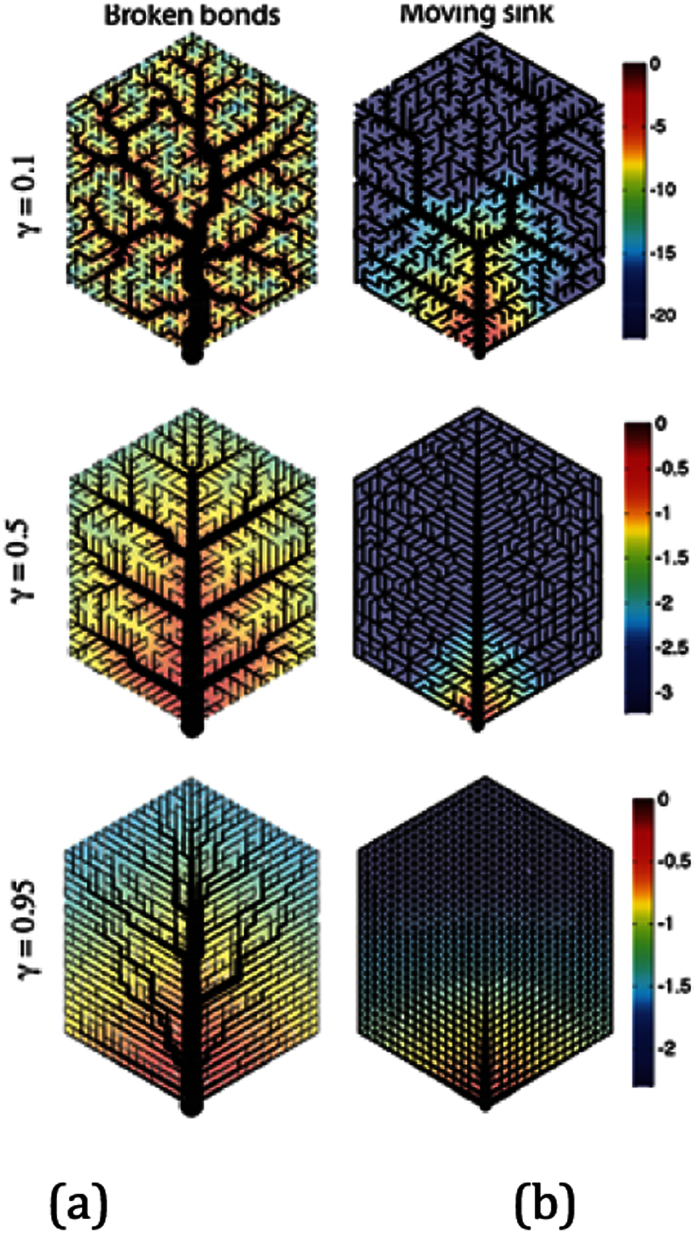
Illustrates the patterning mechanism of two vascular closed looped networks in connection to pressure drop and channel geometry. Fluidic flow will vary in changes in cross sectional channel section within venation network^25^.

A range of electrical potential can determine leaf vasculature optimization by conductance circuit increases. This is a relationship to channel length, radius and hierarchy, Fig. 4.

(a) Defined a network hierarchical multiplication order with a circulative loop network in connection to flow pressure.

(b) Represents a non- hierarchical loop network in relationship to flow pressure.

(c) Colour plot bar denotes fluidic pressure.

(d) Y denotes (dissipation) hierarchical network loop efficiency of flow and conductance distribution.

**Figure 5:**
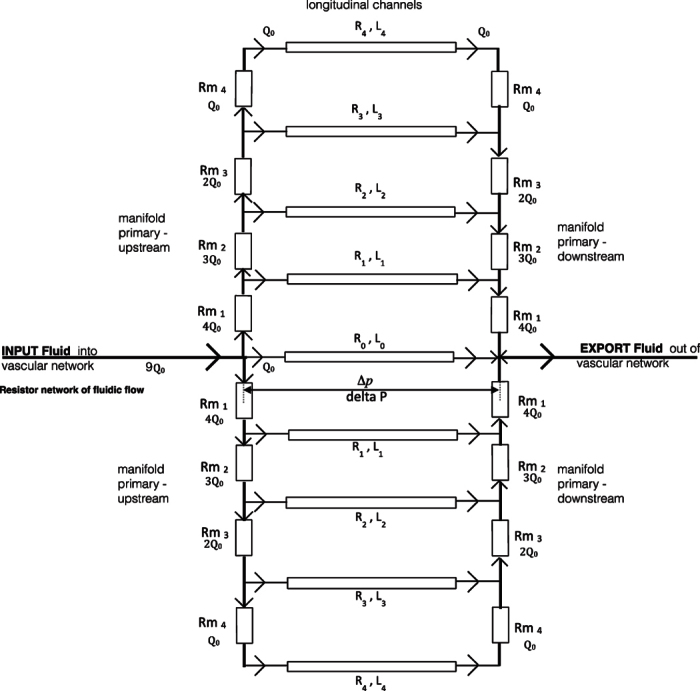
Fluidic Resistor Network.

This is called a sink model fractal network of branching sequential order. Figure 4, denotes the importance of establishing a fixed pressure (or electrical potential) by optimum resistance from fluidic source input. In leaf venation networks this is concentrated in the outermost edges of the leaf of high resistance conductance. Each channel within leaf vasculature is self-organized with its own independency for optimum potential. This hierarchical fluidic transport efficiency for optimal channel networks, Fig. 5, is defined by y = 0.75. When y is small enough, y = 0.25, there is an increasing breaking down of fluidic flow and flow resistance in connection to fluidic source input^12^,^13^. When y > 1.0 the network has no hierarchy with uniform order with nonzero conductance to leaf edges^14^. This represents increased pressure, resistance and concentration of channel cross sectional area focused on fluidic input into the network^15^,^16^. Fluctuations changes in optimum structured networks is a correlationship of laminar fluid flow and resistance^17^. Varying independent channel optimum resistance will determine fluidic transport hierarchy and minimization of pressure drop. This pressure drop will vary resistance in the network between fluidic multi micro channels. To determine pressure drop in longitudinal channels R,L0 to R,L3, Fig. 5, is dependent on upstream (Rp1 to Rp4) and downstream (R_cp1 to R_cp4) micro channels.

Analysis for both the upstream and downstream channels can be determined, to evaluate if the upstream and downstream resistances are different. Knowing the pressure drops (delta P) will allow an estimate of the actual flow resistances. Analysis of delta P will determine optimization of resistance by microchannel sequence succession. Multi micro channels widths are significant as longitudinal microchannels Length and microchannel Depth is determined by material scale. If we assume flow rate is equal within multi microfluidic channels, we can evaluate flow; to predict pressure variation by analysis. Volumetric flow is evaluated by; u_bar (velocity coefficient,) Dh (hydraulic channel diameter), Re (Reynolds number based on hydraulic diameter), Po (Poiseuille number), tau (mean wall channel shear stress) and delta P, along each channel. Resistance is then evaluated from R = delta P/Q flow rate. Fluidic inlet flow to feed distally channels by optimization, is achieved through pressure equalization by diminishing flow pressure variation. This equalization of resistance transport flow is resistance-seeking targeting that can be presented as a resistor network, Fig. 5.

#### Material channel resistor network

Evaluation of resistance optimization is centered on a single microchannel that all other channels succession sequence will emulate. This resistance-seeking targeting is defined as having, R0 and length L0. This microchannel presents the path as least resistance for fluidic flow through the vasculature network. The greatest resistance to flow is presented by R4 as it is distally removed from fluidic input. Other channels are then labeled R1, R2 etc. in sequence from the target resistance channel. If we assume that we know R0 the aim is to design the channel geometry to give equal flow rates through all the microchannels. Fluidic feed in and out flow manifold micro channels are denoted by Rm1, Rm2 etc. Resistances of each longitudinal channel is evaluated and designed to an individual particular resistance function. The equation for the resistances follows a recursive pattern:

Consider the flow channel R1:


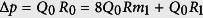


Fluidic flow rate Q, can be cancelled out, as flow rate will be constant, assumed, if equal flow resistance is achieved. Hence analysis of target resistance for the following channels can be considered:


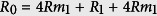



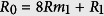



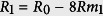


Channel R2:


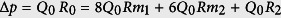



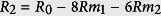


Channel R3:


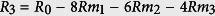


Hence the sequence will continue for R4.

An algorithm is used by increasing the width of the channel in fixed increments of delta w (delta w to be equal to 0.1 microns, to enable 1 micron accuracy). If the resistance of the channel is greater than the “target” hydraulic resistance (of the central channel), then the program increases the width by delta w. Hence a ratio of the maximum resistance to minimum resistance can be optimized. This approach of defining individual channel resistance is the same approach applied in leaf vasculature. This analysis will then automatically feed into calculating the target resistance of the various side upstream and downstream distribution manifold Rm channels. Target resistance is required to give the same flow rate through each channel with the resistance of the outermost channel, R4, needs to be approximately 25% lower than R0. Once optimized widths are achieved the flow rate can be estimated to test whether the proposed method of optimization is successful against CFD simulation in a polymer device.

### Microfluidics Translucent Device Design

The channel vascular geometry design in the polymer device was set at longitudinal channels equal spacing pattern formation of 15.575 mm, with channel widths of: R0–2.0 mm, R1–2.3 mm, R3–2.6 mm, R4–2.8 mm and the outermost channel R4–3.0 mm. Hence target hydraulic resistance of the central channel is determined, as indicated below. This experimentation by an algorithm is a analytical solution for resistance, by channel width succession for sequence optimization.

Hydraulic resistance of central channel (2.0 mm wide × 1.0 mm deep).

Assume a constant temperature of 25 °C. At this temperature, the dynamic viscosity coefficient is equal to


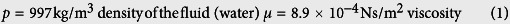


The pressure drop for fully-developed flow along a section of channel of length, *L*, can be determined by balancing the pressure forces and the wall shear forces:


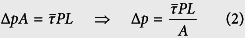


where 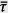 is mean wall shear stress and *P* is the wetted perimeter of the channel, mean flow velocity in the channel.

The Reynolds number of the flow is defined by


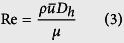


where *D*_*h*_ is the hydraulic diameter of the channel, defined as


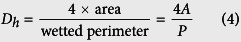


Assuming the flow is laminar, we can then use the Poiseuille number to calculate the average wall shear stress. The mean wall shear stress, 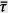, can be related to the *Fanning friction factor*, *f*, which in turn can be expressed as the ratio of the Poiseuille number, Po, and Reynolds number, Re:


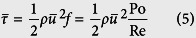


Substituting for Re in Eqn. (5) gives


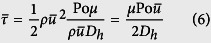


Substituting the shear stress into Eqn. (2) gives


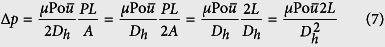


Finally, the *hydraulic resistance*, *R*, of the channel is given by


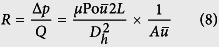
Hence,


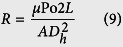


This is identical to Eqn. (18) in Emerson *et al*.^18^.

Consider a 2 mm wide by 1 mm deep channel, 186.243 mm long. The hydraulic diameter of the channel is defined as


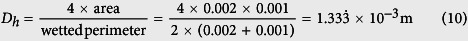


From Table 42 in Shah and London^19^, the Poiseuille number, Po, is 15.54806 for a square duct 2:1 aspect ratio.

Thus,


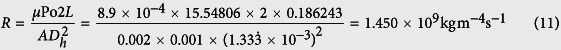


The algorithm follows the same procedures detailed above, with the exception that the Poiseuille number, Po, for a given, channel height and aspect ratio, *α*, is found using the analytical solution involving an infinite series summation (obtained by combining eqns 333 and 340 in Shah and London):


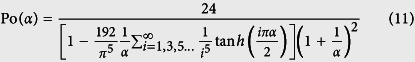


where *α* = *h*/*w*.

Once R0 is known, choosing the optimized value of resistance to achieve equal flow rate through all channels can be determined. This mathematical design procedure predicted pressure drop results of the outermost channel are in good agreement for a fully developed laminar flow that gives validity of the algorithm code. However the ratio of the maximum resistance/minimum resistance = 0.29477840E + 09/0.15429990E + 09 = 1.91. Of the initial channel sequence was high. Resistance variations in the channel sequence are far to high as a optimized solution.

If a fixed pressure was to be applied across the test device, then the flow rates would also vary by the same factor of 1.91. High resistance of the central channel would imply that the volumetric flow rate in the central channel is lower, than that in any of the other channels. The issue is, Fig. 5, the pressure at the start of the longitudinal channels vary considerably by fluidic input channel resistance for the tapered channel sections Rm1 to Rm4.

These distributions input and export manifolds, will change the fluidic pressure to each individual longitudinal channel R0 to R4 entrance and exit. Hence this variation can be predicted in the pressure applied to the individual channels. If the pressure variation in Rm, manifolds channels, is great in comparison to the pressure drop along the channels, the optimization strategy must account for this. Hence the formulated design procedure in terms of the mathematical algorithm can be determined by analysis in a two-stage approach., The second stage program calculates the widths (longitudinal channels) that have been considered in eqns (1, 2, 3, 4, 5, 6, 7, 8, 9, 10, 11, 12).

Determine the widths of the manifold at the location of each longitudinal channel:

The change in manifold width between each successive longitudinal channel is given by


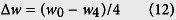


Thus,


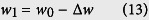



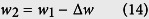



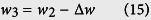


Generalizing:


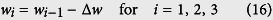


Determine the average width of each of the tapered manifold channels:

(i.e. determine the width of the manifold half-way along each section)


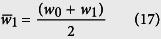



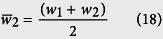



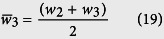



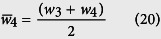


Generalizing:


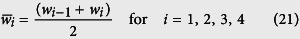


Determine the hydraulic resistances of the manifold channels:

From the previous analysis, we know that the hydraulic resistance, *R*, of a rectangular channel having a constant cross-sectional area is given by


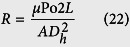


If we assume that the angle of the manifold taper is small, then the individual flow resistances in the manifold can be calculated using the cross-section at the mid-point.

Thus,


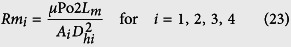


where *A*_*i*_ and *D*_*hi*_ are the area and hydraulic diameter at the mid-point of the manifold channel,

*i*.*e*.


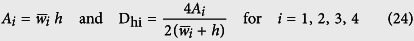


It should be noted that Po in Eqn. (23) is a function of the aspect ratio (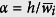) of the channel.

It is informative to calculate the individual pressure drops along the manifold, as these can be checked against CFD results.

Fundamentally, the pressure drop along a section is simply the product of the flow rate and the hydraulic resistance, *i*.*e*. ∆*p* = *QR*. Thus,


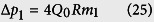



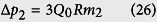



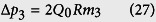



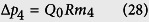


Generalizing:


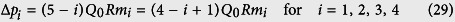


Equation (29) is for the case with four channels either side of the central channel. In the case of *N* side channels:


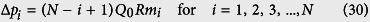


Finally, we calculate the required hydraulic resistances of the longitudinal channels:

The pressure drop across the central longitudinal channel is given by


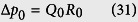


We can then determine the required resistances of the other longitudinal channels:

Consider channel 1:


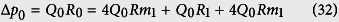


Hence,


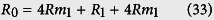


or


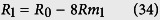


Consider channel 2:


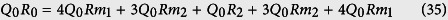


Hence,


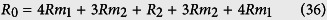


or


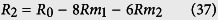


Substituting Eqn. (34) into Eqn. (37) gives


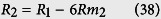


Similarly, it can be shown that


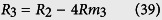


and


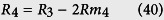


The resistances therefore can be determined recursively using:


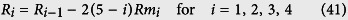


or


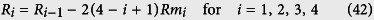


In the case of *N* side channels:


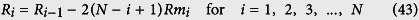


Once we have determined the required resistances, *R*_1_ to *R*_4_, we can then calculate the optimized channel widths. The methodology for this has already been considered to optimize the channels so they each had the same resistance, *R*_0_. An identical procedure is used here but the “target resistance” needs to change slightly for each individual channel to compensate for the additional pressure drop in the distribution manifolds. Flow rates will never be equal to each other, as there are other influences effecting flow rate, such as entrance effects and flow curvature at the start and end of the longitudinal channels. The experimentation analytical solution analysis for the resistance (1–43) requires validation against CFD simulation.

### Computational Fluidic Dynamics–Translucent Device Design

**Figure 6:**
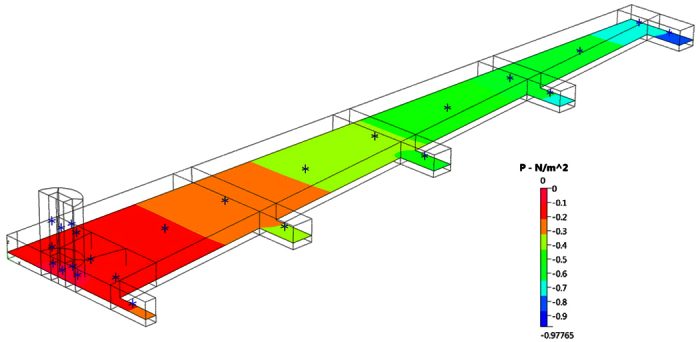
Pressure distribution for input fluid manifold. Symmetry boundary conditions are employed along the centerline of the manifold.

Results of a CFD simulation of the inlet manifold for 2 mm deep set of channels sequences has been undertaken. CFD analysis of the manifold, Fig. 6, focused on the symmetry boundary condition along the centerline of the channel. Prescribing an arbitrary inlet pressure of zero ran the algorithm. A mass flow rate was specified across the outlet boundaries (mdot = 1.66166 × 10^−5^ kg/s) for the full-sized outlets and half this mass flow rate at the central outlet to account for symmetry.

**Figure 7:**
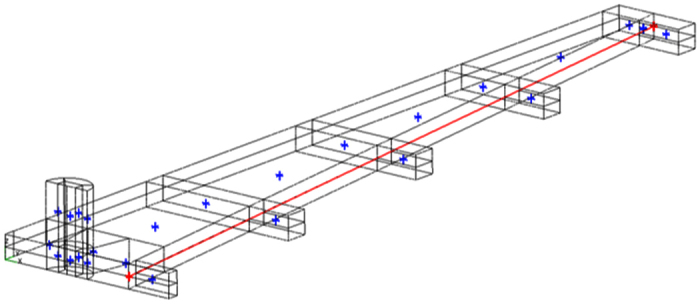
The probe line is located slightly downstream of the manifold so as to highlight the pressures in the channels.

The CFD results show that the pressures at the start of the longitudinal channels vary quite considerably. Hence the pressure at the entrance to the central channel is approximately −0.2 N/m^2^ whereas the pressure at the entrance to the outermost channel is about −0.75 N/m^2^. All pressures are relative to the inlet of the modeled flow polymer device. Hence the pressure difference of 0.55 N/m^2^ exists between the various channels. This compares with a theoretical pressure drop of 4.91 N/m^2^ along the central channel. The pressure drop in the inlet manifold may be up to ~10% of the pressure drops in the main channels. CFD modeling of the device with optimized widths will estimate the flow rate variation between the channels Fig. 7.

**Figure 8:**
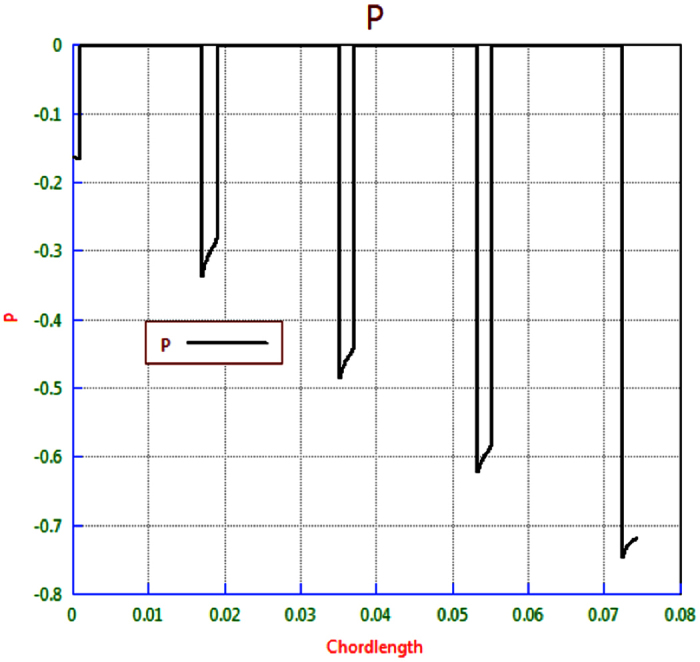
Pressure distribution along the line probe.

CFD simulation gives comparable pressure drops in the four tapered sections and comparable estimates for Rm1 to Rm4. Analysis indicates the approximating the taper by using cross-section, half way along the section is valid for this device. These are the new target resistances; Fig. 8, that gives channel succession steady state flow targets Note that the resistance of the outermost channel, R4, needs to be approximately 25% lower than R0. Second stage is calculating the target resistance of the various channels. Hence the outermost channel R4 was undertaken to assess flow resistance to the target central channel. Below is the CFD simulation of 3.0 mm × 2.0 mm outermost channel:

CFD model data:-

Channel width = 3.0 mm

Channel depth = 2.0 mm

Channel length = 203.635 mm

*ρ* = 997 kg/m^3^ density of the fluid (water)

*μ* = 8.9 × 10^−4^ Ns/m^2^ viscosity of the fluid

A computational mesh composed of 30 × 20 × 400 cells (=240,000 cells) has been used for the CFD simulations.

The volumetric flow rate, *Q*, is specified to be


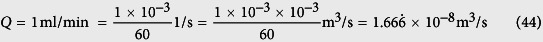


However, the CFD code (CFD-ACE+) needs the flow rate to be specified as a mass flow rate, m in kg/s. Thus,


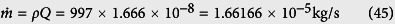


The mass flow rate was specified at the inlet boundary whilst the pressure was set to zero (atmospheric conditions) at the downstream boundary.

Results from the analytical solution:

Input channel width 0.003 (m), channel length 0.203635 (m)

alpha = 0.66667 Po = 14.71183884

u_mean = 0.00277778 m/s

Dh = 0.00240000 m

Re = 7.46816

tau = 0.00757728 N/m^2^

deltap = 2.57167 N/m^2^

Resistance = 0.15429990E + 09

The CFD simulation was setup to estimate the overall pressure drop along the channel for a mass flow rate of 1.66166 × 10^−5^ kg/s (corresponding to 1 ml/min).

**Figure 9:**
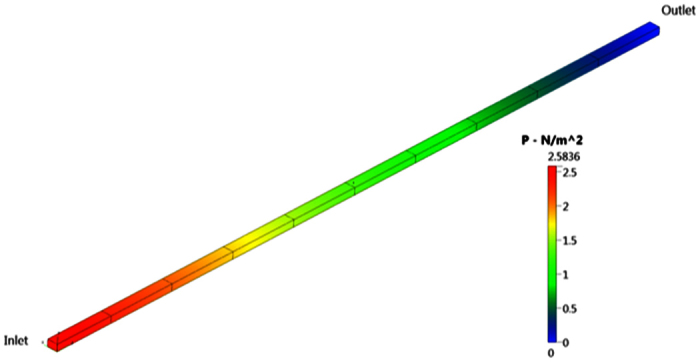
Predicted pressure distribution *R*_4_.

**Figure 10:**
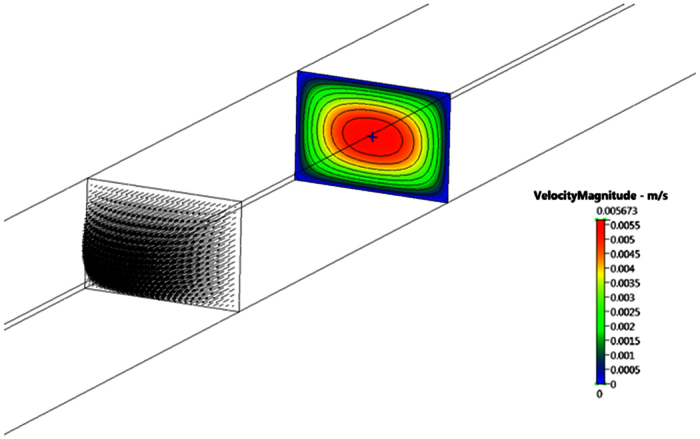
Predicted velocity distribution *R*_4_.

Figures 9 and 10, illustrates that the predicted pressure drop along the channel *R*_4_ is 2.5836 N/m^2^, which is in very good agreement with the analytical solution of 2.57167 N/m^2^.

**Figure 11:**
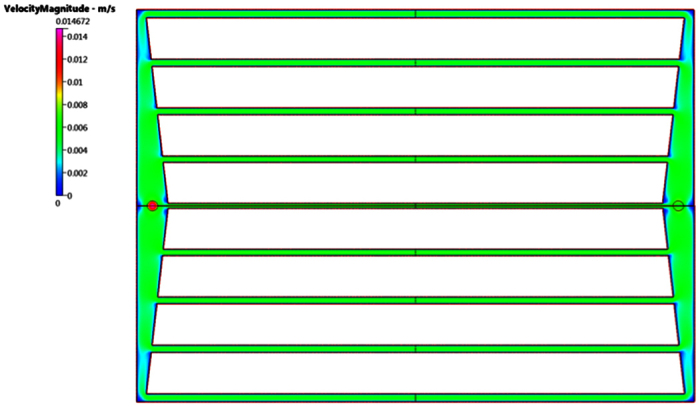
Predicted velocity distribution at a flow rate 9.0 ml/min.

Computations indicate the hierarchical optimized sequence of longitudinal channel widths should be in the following sequential order: R0–2.000 mm, R1–2.095 mm, R2–2.202 mm, R3–2.317 mm and R4–2.431 mm, Fig. 11.

We could also use the CFD results, Tables 1 and 2, to compute the actual hydraulic resistances in the manifold, i.e. Rm1 to Rm4.


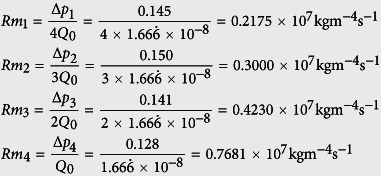


Analytical resistances are generally in good agreement with the CFD results Tables 1 and 2, with a maximum error of ~15% for Rm1, a 7% error for Rm2 and Rm3, and a 1% error for Rm4. This procedure obeys the rules of minimum flow velocity rates, minimum resistance to achieve the lowest pressure drop within a microfluidic network. Each micro channel within this artificial network will influence thermal conductance if subjected to impact heat load. The fluidic medium is therefore acting as a heat sink. Hence fluidic laminar flow rate regulation will determine and influence thermal interface transport exchange to a translucent material device.

### Experimental Testing Method

**Figure 12:**
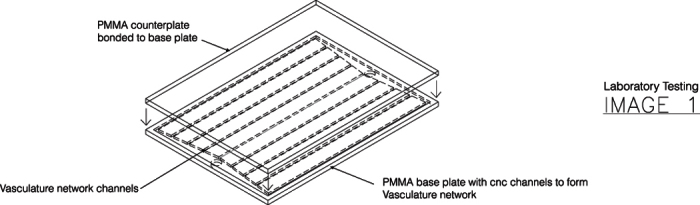
Structural assembly polymer device.

The laboratory testing of the prototype is not focused upon thermal conductivity but the adsorption of solar (ie non-thermal) IR, which then will heat up the polymer structural assembly. Transition temperature of the polymer, will be characterized by volumetric based steady state flow. This capture of energy by solar modulation will progress a thermal function polymer as a IR radiation stop band with lower phase transition temperature. Fabrication consisted of two plates of 5 mm polymer to create the structural assembly. The base plate contained the microchannel network that is fabricated by laser cutting into the surface of the base plate. This channel geometry will contain the microfluidic based flows. The polymer counterplate acts as the solar radiation absorber pane. These two plates have been bonded together to form the structural assembly-testing device, Fig. 12.

**Figure 13:**
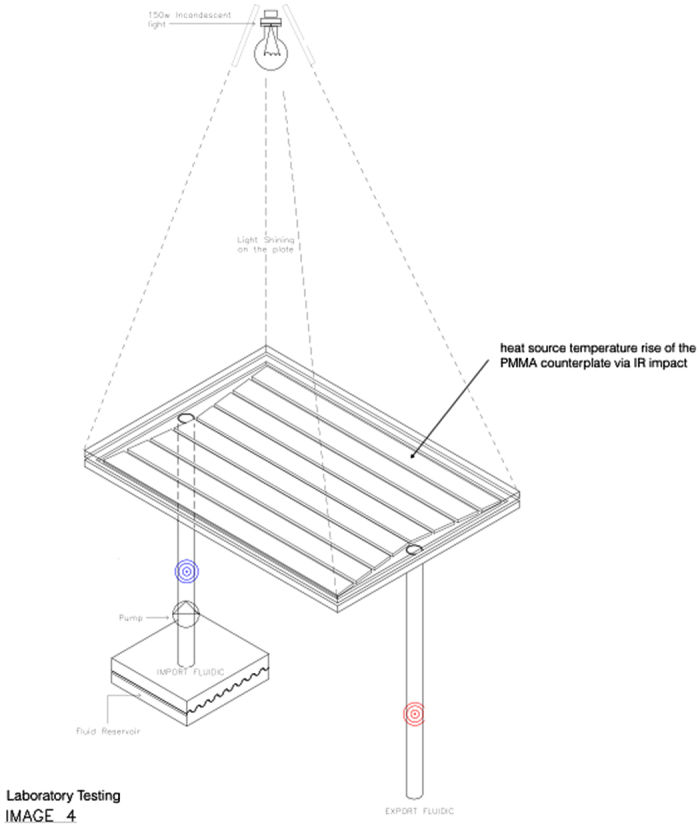
Device Thermal Measurement.

The optically clear polymer is subjected to an artificial solar (incandescent light) source that emitted IR wavelength 1000 watts per m^2^. Solar heat load increase the surface temperature of the polymer surface pane. Distilled water is be pumped through the channel network that directed the structural assembly of the polymer. The fluidic input and extract temperature into the manifolds channels was monitor by thermocouples. Heating of the fluid under flow gave a temperature profile. Sensors monitored material–fluid thermal flow across this interface by extract water temperature. This analysis will enable assessment of thermal switchable IR absorber by water flow. Figure 13 illustrates experimental testing design.

The heating effect from the polymer surface pane and switchability IR absorber was observed by experimentation. Heat transport across the fluid and polymer material interface was evaluated for energy capture. Fluidic interface with polymer material regions, under uniform flow, acted as an IR absorber to lower device phase transition temperature. A load radiation density applied was 1000 W/m^2^ to the device. The counter plate acted as a partially absorbing 210 W/m^2^ pane, the fluid adsorbed 707 W/m^2^ and the remaining 83 W/m^2^ was transmitted by radiation through the polymer. The length of a longitudinal channel in volumetric steady state flow is an individual heat linear absorber of IR within a multi microchannel network. The energy balance of solar radiation is dependent on hydrodynamic behaviour of fluids in steady state pressure driven flows. Results indicated heating of water connected to a partially absorbing pane by passage through the microfluidic based flow gave thermal switching characterization. By modulating volumetric flow rates in the device enabled a temperature difference to decrease roughly inversely with flow rate. Tailored flow rates gave a controlled processing of a thermally functional polymer by microfluidics for desired solar absorber characterization.

### Future Translucent Device Application

**Figure 14:**
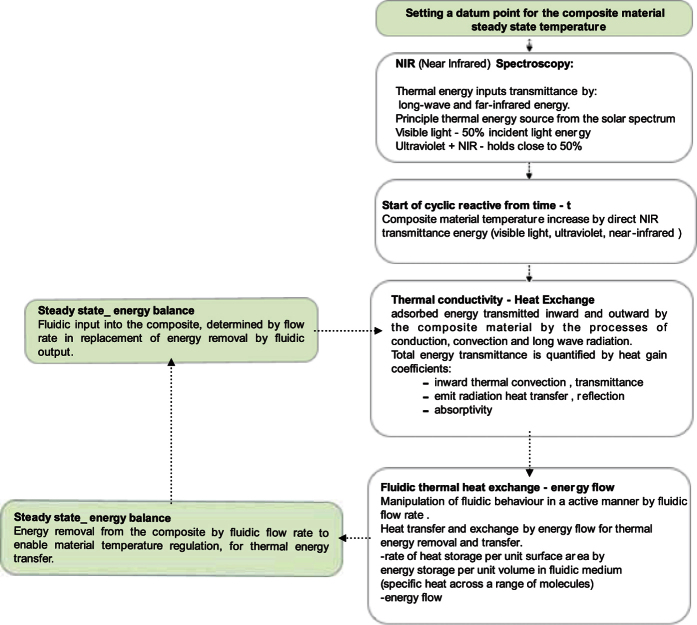
Simulation energy flowchart.

Using microfluidic based flows into a structural assembly of a polymer will advance materials desired energy capture and storage functionality. This steady state flow network of continuously circulating a fluid within it, through it and out of it, by microfluidic based flows to direct the structural assembly of a polymer^20^. This uniform parabolic flow will remove stored liquid temperature out of the polymer for solar energy modulation efficiency. If this liquid is replaced with incoming fluid, this creates a photoabsorptive system. This approach enables thermal switching selectivity of a polymer device in response to heat load, IR. This research is not focused on thermal conductivity but the absorption of solar (non-thermal) IR by heat built up. This represents a thermal exchange transfer cycle of fluidic absorption through vascular channels, Fig. 14.

The micro vascular network will determine thermal switching optimization to material temperature regions. Multi microchannel network will regulate material temperature by management of:

1. Resistance Optimization.
2. Radiative/Convective heat interface transfer.

**Figure 15:**
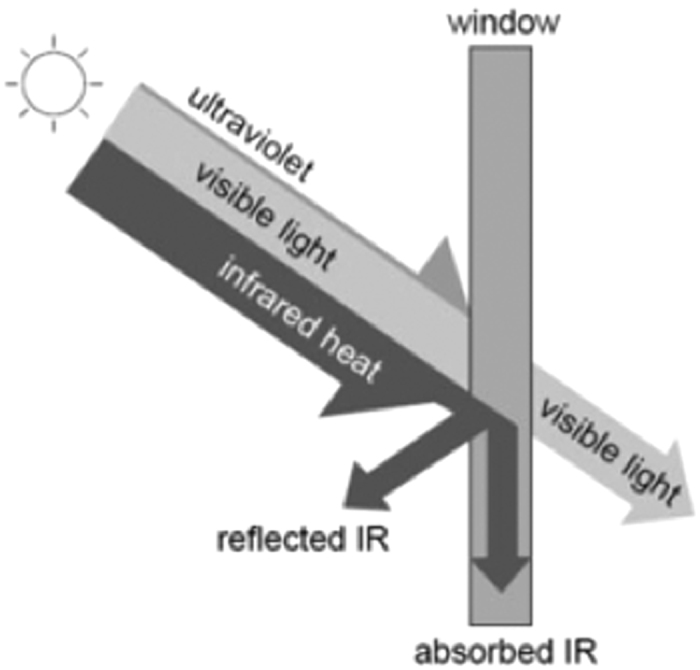
Polymer as an IR block.

These parameters will give optimization of a thermally functional material in relationship to surface temperature fluctuations. The heating effect from a surface material pane is regulated by water uniform parabolic and laminar flow profile for transition temperature decrease, Fig. 15.

Management of thermal flow would progress building translucent facades internal and external surfaces, as the polymer device will act as a thermal flow bridge. Present envelope glazing systems depend on reducing the g-value with solar radiation shading, for minimizing internal thermal load transmission. However these component systems cannot adapt to changing environmental conditions, as they are designed to static boundaries as determined by U-value. These performance modes must change it role from a static element to an energetic façade. Climatic global warming requires performance change by the hour, season and weather conditions. Nature has developed functional materials of complex hierarchy to regulate thermal conductance by venation^21^.

**Figure 16:**
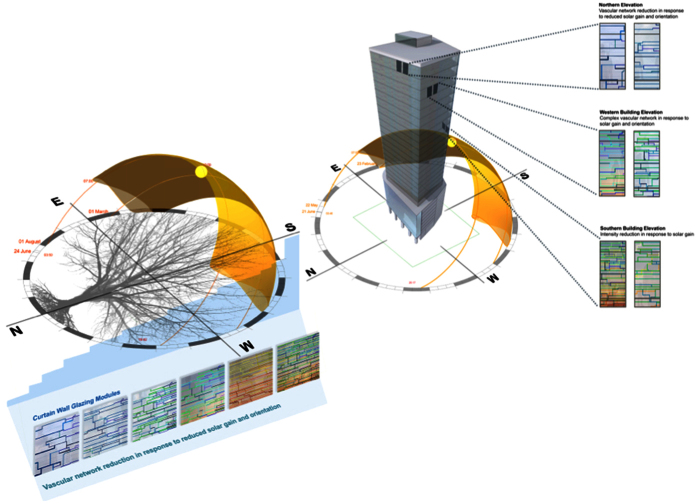
Absorptivity connection to solar orientation.

Greater demands have been placed to minimize operational building energy use by maximizing generated energy and day lighting that are integrated within the building envelope^22^. A thermally functional polymer will advance these aims as a permanent IR absorber, to adapt to changing environmental conditions. To enable transition temperature decrease as a heat flow cycle, for regulation of thermal interface transport exchange to material regions Fig. 16. This thermal management may also enable dehumidification of translucent facades by convective cooling by air to external surfaces.

This is a dynamic heat seeking system to progress current static facade to a thermally functional adaptive layer^23^,^24^. The integration of artificial microfluidic networks of solar absorbing fluid in active flow is a new methodology. Future progression is to determine thermal characterization of the device by thermal switching for heat flow targeting. This may impact on the vascular resistor computational process via measuring thermal conductance heat flow fluxes, in relationship to laminar thermal flow absorption.

### Conclusion of Methods

This iterative procedure will determine optimization of vasculature employing resistance seeking targeting by hierarchical channel succession, as a resistor networks. Hence this optimization process is determined by selection of a known hydraulic resistor value *R*_0_, to enable evaluation that determines vascular channel network geometry, to give flow equalization as a development mechanism as denoted by:


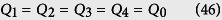


The pressure drop across the outermost longitudinal channel is given by


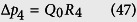


We can then determine the required resistances of the other longitudinal succession channels *R*_1_, *R*_2_, *R*_3_ etc that can be determined as a recursive pattern:


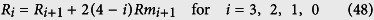


Feed in principle upstream and down stream channels, manifold resistance, *Rm*_1_, *Rm*_2_, *Rm*_3_ is determined by *N* side channels (*N* denoting the total number of channels is 2*N* + 1):


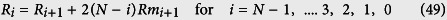


This computation design methodology of the device predicted pressure drops results in the manifold are in good agreement with CFD results. Results indicate resistances of the longitudinal microchannels are similar to the theoretical results. The tapered sections resistances are in good agreement, except for the tapered section involving the inlet port. This is expected since the computation solution cannot take into account the flow expanding away from the circular inlet port.

Computational analysis conclusively demonstrates being able to design microfluidic networks using a theoretical approach, to achieve optimization of circuit resistance of transport fluidic flow. Optimization is achieved through pressure equalization by diminishing flow pressure variation. The two-step algorithm approach enables self-organized channel resistance with its own independency for optimum potential for pressure drop regulation. Methodology of successive conduit sequences of hierarchical formations perform regulatory roles. This is functionally significant in the analysis of hydraulic resistance to compute simulations of flow rates in microvasculature channel networks. Predicted pressure drop and flow analysis within channel network is in agreement with the analytical solution for fully-developed laminar flows, giving validity to the algorithm code as a iterative procedure.

The analytical results using theoretical resistance are based on R0. The weakness of this approach is represented by R1 and R0, as CFD simulations already indicate that flow rates through R4, R3 and R2 are almost identical. If the network is constrained to device 148 mm width, multi microchannel sequence succession could start from the outermost channel and work inwards for R3, R2, R1, and R0. The attraction of this is the footprint of the network stays constrained to a 148 mm width with the outermost edge 3 mm microchannel. This method is a reverse analysis as the outermost channel width is unknown to begin with and so the width of the device cannot be known in advance. The optimized resistance condition could be based on theoretical manifold resistance. If design channel widths uses Rm manifold resistance obtained from CFD simulation to estimate the desired flow resistance in longitudinal channels, as given by:


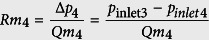


Flow rate through the manifold is denoted by *Qm*. This would lead to greater optimized microfluidic design, by code modification to obtain uniform flow across the polymer pane. The morphogenesis of leaf vasculature sets an underlying process of flow distribution, pressure, fluidic transport and resistance. This notion of precise hydrodynamic control of microfluidic’s will progresses thermal material characterization; to advance a polymer device, into a switchable IR absorber. This is achieved by hierarchical succession of branching sequence patterns that conform to rules of minimum effective power flow rates in the transportation of fluidics within fractal networks. That is determined by computational theoretical analysis of resistance seeking targeting.

## Additional Information

**How to cite this article**: Alston, M. E. and Barber, R. Leaf venation, as a resistor, to optimize a switchable IR absorber. *Sci. Rep.* **6**, 31611; doi: 10.1038/srep31611 (2016).

## Figures and Tables

**Figure 1:**
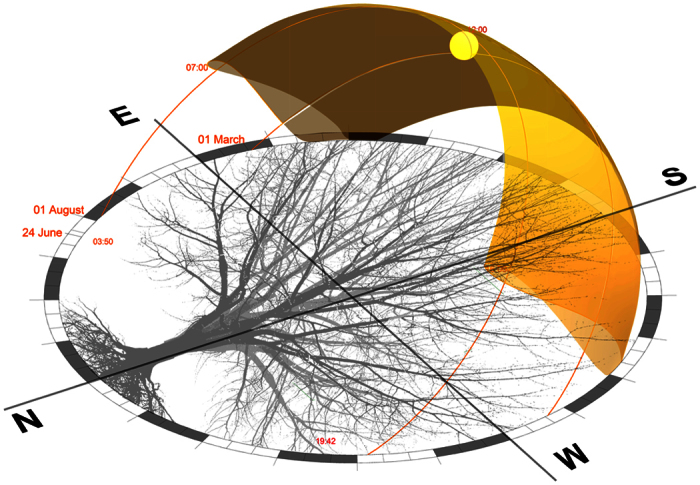
Tree structural geometry in relationship to solar orientation.

**Figure 2:**
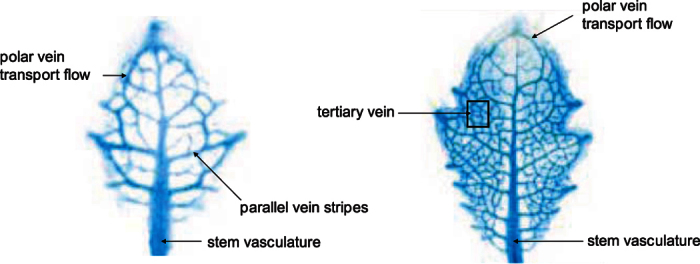
Leaf Vasculature Formations.

**Table 1:** CFD results: pressure distribution in the manifold for a flow rate of 1 ml/min through each outlet channel (0.5 ml/min through central channel due to flow symmetry).

Channel #	P_left(N/m^2^)	P_right(N/m^2^)	P_average(N/m^2^)	Deltap relative toprevious channel (N/m^2^)
0	0.163	0.160	0.162	—
1	0.334	0.278	0.306	0.145
2	0.478	0.434	0.456	0.150
3	0.616	0.577	0.597	0.141
4	0.732	0.717	0.725	0.128

**Table 2:** Analytical solution for the pressure channel distribution.

Channel #	Delta p relative to previous channel (N/m^2^)
0	—
1	0.167
2	0.161
3	0.151
4	0.127
